# Genome Sequence of the Hepatitis C Virus Subtype 6n Isolated from Malaysia

**DOI:** 10.1128/genomeA.00168-12

**Published:** 2013-02-14

**Authors:** Kim Tien Ng, Yeat Mei Lee, Haider Abdulrazzaq Abed Al-Darraji, Xueshan Xia, Yutaka Takebe, Kok Gan Chan, Ling Lu, Sanjiv Mahadeva, Adeeba Kamarulzaman, Kok Keng Tee

**Affiliations:** a Centre of Excellence for Research in AIDS (CERiA), Department of Medicine, Faculty of Medicine, University of Malaya, Kuala Lumpur, Malaysia; b Faculty of Environmental Science and Engineering and Faculty of Life Science and Technology, Kunming University of Science and Technology, Kunming, Yunnan, China; c AIDS Research Center, National Institute of Infectious Diseases, Toyama, Shinjuku-ku, Tokyo, Japan; d Division of Genetics and Molecular Biology, Institute of Biological Sciences, Faculty of Science, University of Malaya, Kuala Lumpur, Malaysia; e Viral Oncology Center, Department of Pathology and Laboratory Medicine, University of Kansas, Medical Center, Kansas City, Kansas, USA; f Department of Medicine, Faculty of Medicine, University of Malaya, Kuala Lumpur, Malaysia

## Abstract

We report the full genome sequence of hepatitis C virus (HCV) subtype 6n from Kuala Lumpur, Malaysia. Phylogenetic analysis of the isolate 10MYKJ032 suggests that Southeast Asia might be the origin for the HCV subtype 6n and highlights the possible spread of this lineage from Southeast Asia to other regions.

## GENOME ANNOUNCEMENT

Hepatitis C virus (HCV), a positive-sense RNA virus from genus *Hepacivirus* of the family *Flaviviridae*, is a predominant and widely distributed bloodborne pathogen associated with liver cirrhosis and hepatocellular carcinoma in humans (1). According to the World Health Organization, HCV infects approximately 150 million people worldwide (2), of which 10 million are injecting drug users (3). To date, there are six confirmed genotypes, denoted genotypes 1 to 6 (4), and a provisionally assigned genotype 7 (5), with each genotype diverging further into multiple subtypes with distinct genetic variability. Such genetic diversity has been significantly associated with geographical and epidemiological factors (4, 6, 7). Genotypes 1, 2, and 3 are distributed globally; genotype 4 is confined to the Middle East and Central Africa; genotype 5 is predominantly found in South Africa; and genotype 6, the most divergent genotype, is largely found in Southeast Asia (8, 9). Here, we describe the first genome sequence of HCV subtype 6n isolated in Kuala Lumpur, Malaysia, from a consenting 37-year-old HIV-1-infected homosexual male with a history of injecting drug use.

A plasma sample was collected in July 2010, and HCV RNA was extracted using the NucliSENS easyMAG automated nucleic acid extraction system (bioMérieux, Marcy I’Etoile, France), reverse-transcribed, and amplified using sets of newly designed and previously published primers (10) spanning the complete genome of HCV that encodes the structural proteins (C, E1, and E2), nonstructural proteins (P7, NS2, NS3, NS4A, NS4B, NS5A, and NS5B), and the 5′- and 3′-untranslated regions (UTRs). The contiguous nucleotide sequences generated by an ABI Prism 3730xl DNA analyzer (Applied Biosystems) were assembled to generate a near-full-length genome of 9,403 bp with an intact open reading frame. Codon alignment was generated with a comprehensive list of reference sequences retrieved from the HCV database (http://www.hcv.lanl.gov/). Maximum likelihood inference was performed using Phylogenetic Analysis Using Parsimony (PAUP) (11), with a Hasegawa-Kishino-Yano nucleotide substitution model and gamma distribution plus discrete gamma categories. In this model, the maximum likelihood phylogenies were heuristically deduced using subtree pruning and regrafting, as well as nearest neighbor interchange algorithms. The statistical robustness and reliability of the branching orders were evaluated by a bootstrap analysis of 1,000 replicates. A neighbor-joining tree was also reconstructed based on the Kimura 2-parameter model implemented in Molecular Evolutionary Genetics Analysis (MEGA) version 5.05 (12).

In the present analysis, both maximum likelihood and neighbor-joining phylogenies showed that the full genome of isolate 10MYKJ032 formed a monophyletic cluster with previously described HCV subtype 6n, namely, isolates KM42 from China (13) and D89/93, TH22, and TH31 from Thailand (10, 14). More remarkably, isolate 10MYKJ032 was located at the root of the 6n lineages with strong statistical support, suggesting its ancestral or parental relationships with other reported HCV 6n genomes. Together with the Thai isolates, phylogenetic inference showed that Southeast Asia may be the plausible geographical origin of HCV subtype 6n. Furthermore, spatial analysis indicated possible migration of HCV 6n lineages from Southeast Asia to the southern regions of China. Such genetic information is important to understand better the evolutionary behaviors of HCV subtype 6n and to trace disease spread in the region.

### Nucleotide sequence accession number.

The genome sequence of 10MYKJ032 has been deposited in GenBank under the accession no. KC191671.
